# Intracellular ATP Delivery Causes Rapid Tissue Regeneration *via* Upregulation of Cytokines, Chemokines, and Stem Cells

**DOI:** 10.3389/fphar.2019.01502

**Published:** 2020-01-16

**Authors:** Yiqun Mo, Harshini Sarojini, Rong Wan, Qunwei Zhang, Jianpu Wang, Sarah Eichenberger, Girish J. Kotwal, Sufan Chien

**Affiliations:** ^1^ Department of Environmental and Occupational Health Sciences, School of Public Health and Information Sciences, Louisville, KY, United States; ^2^ Department of Surgery, School of Medicine, University of Louisville, Louisville, KY, United States; ^3^ Noveratech LLC, Louisville, KY, United States

**Keywords:** ATP-vesicle, wound, rabbit, cytokine, growth factor

## Abstract

We have reported accelerated wound healing induced by intracellular ATP delivery in rabbits, through early massive accumulation, *in situ* proliferation, and M2 polarization of macrophages. Granulation tissue started to grow within first 24 h of treatment and continued the growth till the wound cavity is completely covered. However, the mechanisms underlying this macrophage response are totally unclear because no one has ever reported this before. In this study, we performed a preliminary exploration of the possible mechanisms by focusing on the roles of cytokines, growth factors, and stem cells in this process. Among the 33 adult rabbits, 18 were used for cytokine measurements and the remaining were used for histological and immunohistochemical studies. Four wounds were created on the ventral side of each ear. Two wounds on one side were treated with ATP-vesicles (10 mM ATP), and the other two were treated with controls (normal saline or Regranex). Dressing changes were made daily and the rabbits were sacrificed at 5 h, 12 h, and 1, 2, 3, 4, 6, 9, 15, and 26 days after wounding. Tissue samples were analyzed for cytokines and growth factors using real-time PCR and immunohistochemical staining. The control wounds showed an immediate increase in proinflammatory cytokines after wound creation but no further increase after this initial spike. The growth factor levels in the control wounds remained unchanged throughout the study. Conversely, the wounds treated with ATP-vesicles showed significantly higher expression of MCP-1 and stem cell markers (CD44, CD106, CD146, and CD34) at day 1, significantly higher IL-1β and TNF-α expression from day 1–4, and significantly higher VEGF-A, VEGF-D, and VEGFR-2 expression from day 4–6 when compared to the controls. The significant upregulation of these factors corresponded to the very early and rapid macrophage accumulation, *in situ* proliferation, and M2 polarization, resulting in unprecedented rapid granulation tissue generation due to direct macrophage collagen production and neovascularization.

## Introduction

Wound healing is a multifaceted physiological process in reaction to tissue damage. Numerous factors are involved in the wound healing process; however, for many chronic wounds, the primary pathology of a non-healing wound is a deficient blood supply or tissue ischemia, leading to the long recognized observation that “wounds do not heal in tissue that do not bleed, whereas they almost always heal in tissue that bleeds extensively” (Gottrup, 2004).

The process of wound healing requires cellular energy in the form of ATP for every phase; thus, any decrease in the availability of ATP negatively impacts every aspect of the healing process (Smith et al., 1999). A logical hypothesis, therefore, is that delivery of ATP to the cytosol of wound cells will be beneficial to the healing process. We previously demonstrated that treatment of full-thickness skin wounds in rodents and rabbits with ATP-encapsulated lipid vesicles (ATP-vesicles) for intracellular delivery of ATP accelerated wound healing process when compared with vehicle (lipid vesicles only), normal saline, or Regranex, the only FDA-approved prescription growth factor for wound care (Howard et al., 2014). At the same time, we also noted a phenomenon: the appearance of granulation tissue within 24 h after treatment. This granulation tissue had covered most of the entire wound area by day 5 in many wounds. By contrast, the control wounds treated with the other dressings showed little or no granulation tissue until day 6 or 7. This rapid granulation tissue growth has never been reported previously in humans or any other land animals, nor has it been observed with any other drugs or therapeutics. Further histologic and immunohistochemistry studies revealed that the most prominent response to ATP-vesicles was the extremely early and rapid accumulation and *in situ* proliferation of macrophages that filled the most portion of the newly formed granulation tissue (Howard et al., 2014; Sarojini et al., 2017). However, the mechanisms underlying such a rapid macrophage reaction were totally unclear.

Activated macrophages and the cytokines they release are recognized as having a vital role in wound healing, and the progression of macrophage trafficking, proliferation, and polarization is known to be mediated by locally released growth factors and cytokines, which may act in an autocrine or paracrine manner (Marisa et al., 2005). The aim of the present study was therefore to examine the mechanisms underlying the extremely rapid accumulation and *in situ* proliferation of macrophages in response to intracellular energy delivery by focusing on several key cytokines, chemokines, and growth factors that are correlated with rapid wound healing.

## Materials and Methods

### Preparation of ATP-Vesicles

The ATP-vesicle formulation was prepared by Avanti Polar Lipids, Inc. (Alabaster, AL). The composition was 100 mg/ml of Soy PC/DOTAP (50:1), Trehalose/Soy PC (2:1), 10 mM KH_2_PO_4_, and 10 mM Mg-ATP. The diameters of the lipid vesicles ranged from 120 to 160 nm. The lyophilized ATP-vesicles were stored at −20°C and were reconstituted and mixed with a nonionic vanishing Velvachol cream immediately before use (Chiang et al., 2007; Wang et al., 2010). Regranex or normal saline were used as controls. Previous studies on the hypoxia induced cell survival by ATP-vesicles and their individual components (Mg-ATP alone, lipid vesicles alone, and Mg-ATP plus lipid vesicles) showed cells were protected from hypoxia only by ATP-vesicles treatment (Chien, 2010a; Chien, 2010b). We tested all the components in cell culture before animal studies. None of the individual components, including the empty lipid vesicles, free Mg-ATP, or free Mg-ATP plus empty lipid vesicles (without encapsulation) had the same effect as of ATP-vesicles. (Chiang et al., 2007; Chien, 2010; Chien, 2010).

### Animals Used

Animal use was reviewed and approved by the University of Louisville Institutional Animal Care and Use Committee. Thirty-three adult New Zealand white rabbits (1.5–3.0 kg, Myrtle’s Rabbitry, Thompson Station, TN; and Charles River Laboratories, Cleveland, OH) were used in this study. They were divided into the following groups:

1. For histology and immunohistochemistry study: Fifteen rabbits (72 wounds) were used in which ATP-vesicles, Regranex, or normal saline were applied as dressings. These rabbits were sacrificed at different times after surgery.
2. For cytokine study: Eighteen rabbits were used in which ATP-vesicles and normal saline were used as dressings and the rabbits were sacrificed at different times after surgery.

### Wound Creation

Wound creation and healing comparisons were conducted as reported before (Chien, 2007). Briefly, each rabbit was weighed and anesthetized with a mixture of ketamine (50 mg/kg) and xylazine (5 mg/kg) intramuscularly. Both ears were shaved, prepared aseptically, and draped so that both ears were exposed. On the ventral side of each ear four full-thickness circular skin wounds were created with a 6-mm stainless steel punch. Distance among each wounds was at least 30 mm. The skin inside the punch wound was removed from the cartilage. The perichondrium was also removed with or without the skin. The granulation and epithelialization took place over the cartilage, and the cartilage was not punctured.

### Postoperative Management

Postoperatively, a Duragesic patch was attached to the back skin to release Fentanyl (25 µg/h) for 2–3 days for reduction of possible pain. The rabbits were allowed free access to water and food. ATP-vesicles (10 mM ATP) standardized dose as reported (Howard et al., 2014; Sarojini et al., 2017) earlier were applied to two wounds on one side of each ear, and the other two wounds were treated with control dressings (Regranex or normal saline). The wounds were then covered with an occlusive dressing (TegaDerm™, 3M, Minneapolis, MN). Dressing changes were made daily and digital photos were taken of all wounds. For histology, immunohistochemistry, and cytokine studies, the animals were sacrificed postoperatively at 5, 12h, and 1, 2, 3, 4, 6, 9, 15, and 26 days, and the wound with 2–3 mm of surrounding tissues were removed. One part of the sample tissue was frozen in liquid nitrogen immediately and then stored in a −80 °C freezer for later total RNA extraction. Another part was fixed in 10% buffered formalin and embedded in paraffin for histologic and immunohistochemistry studies.

### Isolation of Total RNA From Wound Tissues

TRI Reagent (Sigma, St. Louis, MO, USA) was used to isolate total RNA from rabbit wound tissues. Briefly, wound tissue was homogenized in 1 ml of TRI Reagent using a power homogenizer (Glas-Col, Terre Haute, IN). After incubation for 5 min at room temperature, 0.2 ml of chloroform was added per ml of TRI Reagent and the solution was mixed vigorously for 15 s and was left to stand at room temperature for another 10 min. After centrifugation a colorless upper aqueous phase was collected, and total RNA was precipitated by mixing with 0.5 ml of isopropyl alcohol per ml of TRI Reagent. The RNA pellet was dissolved in RNase-free water after washing with 75% ethanol. The RNA concentration was measured as absorbance at 260 nm with a DU 730 Spectrophotometer (Beckman Coulter, Fullerton, CA).

### Reverse Transcription and Real-Time PCR

Reverse transcription (RT) and real-time PCR were performed to detect the mRNA expression levels of MCP-1, IL-1β, and TNF-α in wound tissues. Briefly, 2 µg total RNA was reverse-transcribed into cDNA using 1µl M-MLV reverse transcriptase (Promega, Madison, WI) in a total volume of 25 µl containing 2 µl 0.5 µg/µl oligo(dT)_18_ primer, 1.25 µl 10 mM dNTP, 0.75 µl RNasin^®^ Ribonuclease inhibitor, 5 µl 5 × M-MLV reaction buffer. A 1 µl volume of cDNA solution was used to perform real-time PCR on an iQ5 multicolor real-time PCR detection system (Bio-Rad, Hercules, CA) using an iQ SYBR Green Supermix kit (Bio-Rad). Briefly, 1 µl cDNA from each sample was mixed with 1 µl of each primer (5 µM) and 10 µl 2× SYBR Green Supermix in a total volume of 20 µl. The experimental protocol consisted of four programs: 1) denaturation of the cDNA/RNA hybrid at 95°C for 3 min; 2) amplification of cDNA for 50 cycles, each cycle using sequentially 95°C for 10 s, 58°C (MCP-1, IL-1β and TNF-α) or 59.5°C (GAPDH) for 30 s, and 72°C for 30 s; 3) analysis of the melting curve to confirm the single product amplification during the PCR assay; and 4) cooling the rotor and thermal chamber at 25°C. The following primers were used for real-time PCR: for rabbit GAPDH (507 bp), 5’-ATG TTT GTG ATG GGC GTG AAC C-3’ and 5’-CCC AGC ATC GAA GGT AGA GGA-3’; for rabbit MCP-1 (230 bp), 5’-TGT GCT TGC CCA GCC AGA TG-3’ and 5’-GTG TCT GCA TTT TCT TGT CC-3’; for rabbit IL-1β (358 bp), 5’-GCA CCT CTC AGA CAG AGT AC-3’ and 5’-GTG GTT GCT GAT AGA AGC TG-3’; and for rabbit TNF-α (436 bp), 5’-CAA GCC TCT AGC CCA CGT A-3’ and 5’-GGC AAT GAT CCC AAA GTA G-3’. The relative expression level of each gene was calculated as fold dilution using a standard curve for each gene. Standard curves were obtained by real-time PCR using 3.0, 1.0, 0.1, 0.01, and 0.001 µl dilutions of cDNA obtained from normal rabbit skin. The expression level of each gene was then normalized with the relative expression level of rabbit GAPDH in the same sample.

### Immunohistochemical Staining

Formalin-fixed tissues, processed with the Thermo Fisher Path Centre (Fisher Scientific, Chicago, IL), were embedded in paraffin blocks and cut into 5 µm sections. One set was stained with hematoxylin and eosin (H & E) for general histological evaluation. Platelet infiltration was detected by immunohistochemical staining using anti-ITGB3 (CD61) antibody (My BioSource, San Diego, CA). Anti-MCP-1 antibody (Abcam, Cambridge, MA) was used to detect the presence of chemoattractants for monocytes. CD44, VCAM (CD106) (Abcam, Cambridge, MA) and CD146 (Biorbyt, San Francisco, CA) antibodies were used to detect the presence of mesenchymal stem cells (MSCs). Anti-CD45, anti-collagen type I (Abcam, Cambridge, MA), anti-CD34 (Dako, Carpinteria, CA), anti-VEGF-D (R&D Systems Inc., Minneapolis, MN), anti-VEGF-A (Santa Cruz, Dallas, TX), anti-VEGF-R2 (Santa Cruz, Dallas, TX), and anti-VEGF-R1 (Santa Cruz, Dallas, TX) primary antibodies were used to study neovascularization. Histology images were analyzed with a Nikon Eclipse Ti microscope (Nikon Instruments, Inc.). The captured images were further quantified morphometrically using Nikon Elements Imaging software. For quantification of immunohistochemical staining, the number of positive cells and total cells per high power microscopic field were counted, for at least 5 fields for each slide. The number of positively stained cells was expressed as a percentage of the total number of cells.

### Statistical Analysis

Values were expressed as mean and standard deviation. Two-way analysis of variance (ANOVA) were used to evaluate the differences among groups followed by Dunnett’s test. A *p* value less than 0.05 was considered statistically significant. Statistical analyses were carried out using Microsoft excel or SigmaPlot software (Systat Software, Inc., San Jose, CA, USA).

## Results

### Rapid Tissue Generation

**Figure 1:**
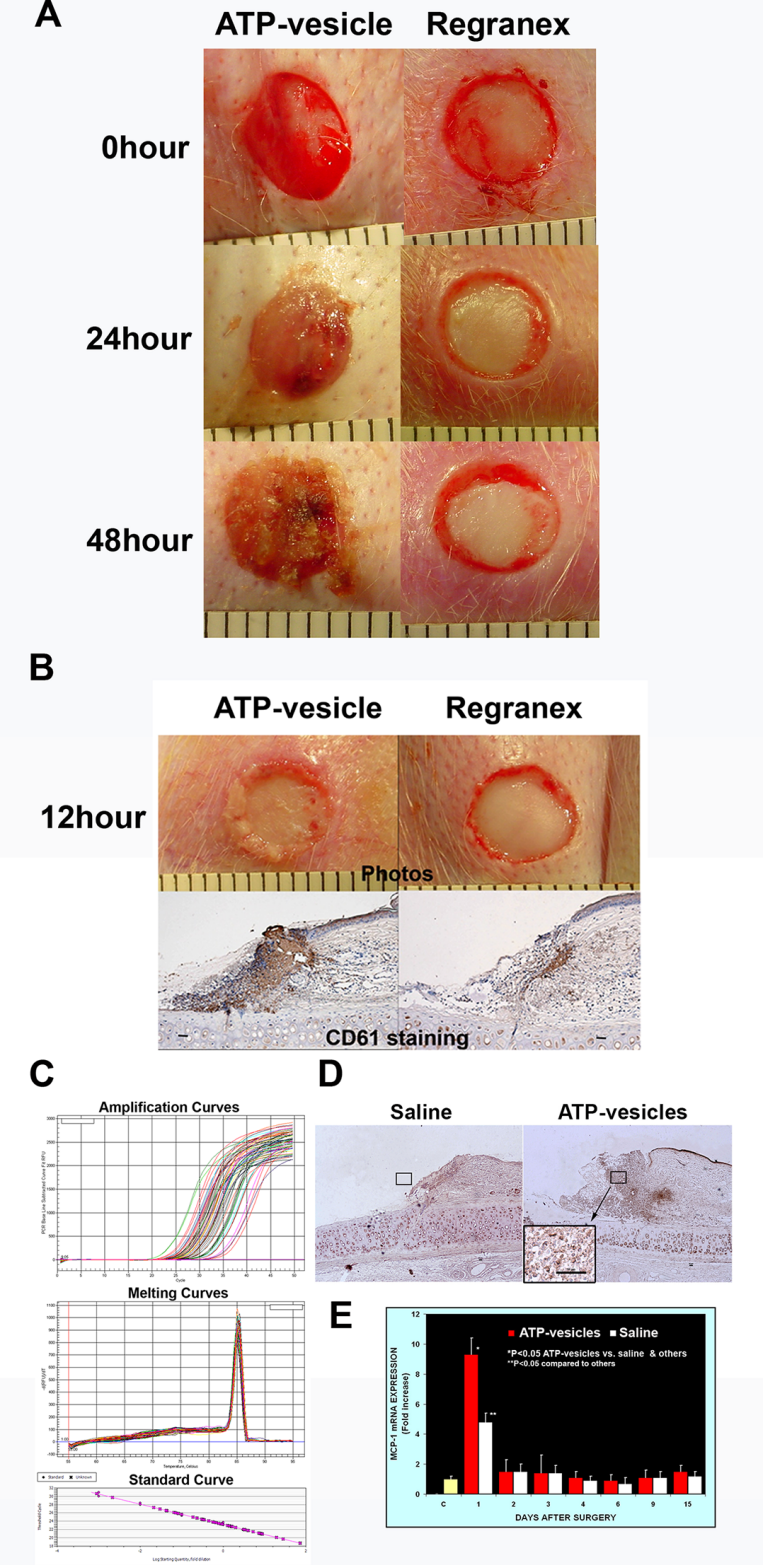
**(A)** Comparison of early tissue growth in wounds treated with ATP-vesicles and Regranex. Granulation tissue already started to grow within 24 h in the ATP-vesicle–treated wounds while no growth was seen in Regranex-treated wound. **(B)** Anti-CD61 staining of these wounds shows the presence of platelets with a brown color (scale bar = 50 μm) as early as 12 h of ATP-vesicle treatment. But almost no accumulation of platelets was observed in Regranex-treated wounds. Platelets are the first responders at the wound site for initiating the inflammation and healing process before granulation tissue formation. Note: Regranex is a recombinant human platelet-derived growth factor, so it shows some immunoreactivity with CD61 in the control wounds. **(C** and **E)** Comparison of MCP-1 expression in wounds from day 1 to day 15 post surgery. Real-time PCR was performed with an IQ5 Cycler (Bio-Rad). Data shown are the mean ± SD of four to six different wound tissues, with triplicate assays for each sample. Normal rabbit ear skin was used as a baseline control (C). *Significantly different when compared with both the saline treated control group and the baseline control, p < 0.05. **Significantly different when compared with the baseline control only, p < 0.05. **(D)** Immunohistochemical comparison of wounds treated with ATP-vesicles and normal saline. Anti-MCP-1 immunoreactivity was higher in the ATP-vesicle–treated wounds than in the saline–treated wounds. The brown color indicates positive immunostaining (scale bar = 50 μm).

The most striking finding was the extremely rapid tissue generation following application of ATP-vesicles: In most ATP-vesicle–treated wounds, gross granulation tissue started to appear within 24 h after surgery (**Figure 1A**). Another major result in this study was very early and quick platelet accumulation. We examined the role of platelets using anti-ITGB3 (CD61) antibody to detect both platelets and platelet microparticles. We were able to detect the presence of platelets in ATP-vesicle–treated wounds as early as 12 h post treatment (**Figure 1B**). By contrast, the control wounds treated by Regranex showed much less platelet accumulation. Platelets are normally the first responders at the wound site for initiating the inflammation and healing process, but treatment of ATP-vesicles significantly enhanced their trafficking and accumulation.

### Cytokine Production

**Figure 2:**
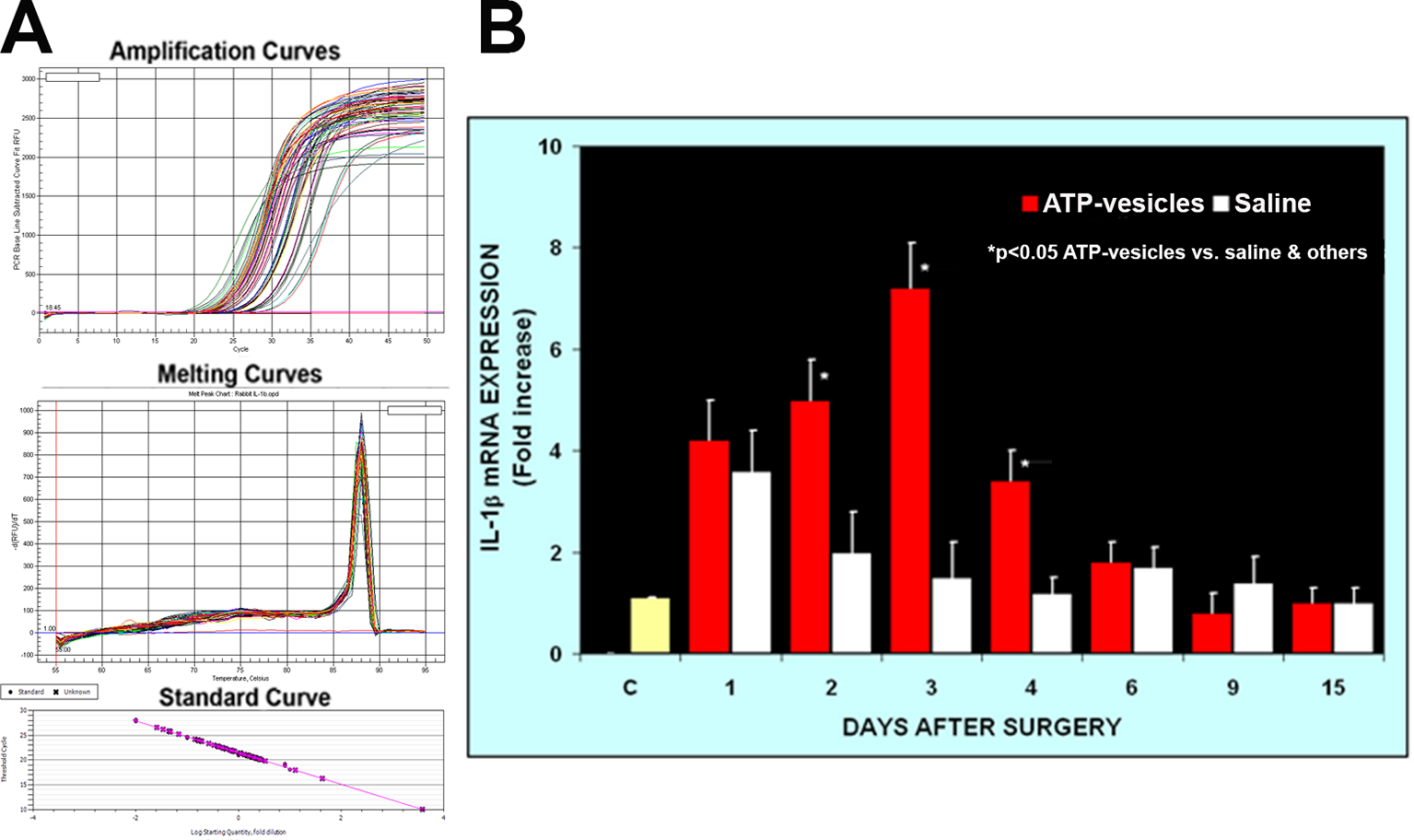
Real-time PCR comparison of IL-1β expression in wound tissues of ATP-vesicle–treated (study) and the saline treated (control) groups. **(A)** represents the amplification curves, melting curves, and standard curve of real-time PCR and **(B)** shows their quantitative fold changes indicating higher expressions from day 1 to day 4 by ATP-vesicles. Data shown are the mean ± SD of four to six different wound tissues, with triplicate assays for each sample. Normal rabbit ear skin was used as a baseline control (C). *Significantly different when compared with saline-treated control group and the baseline control, p < 0.05.

**Figure 3:**
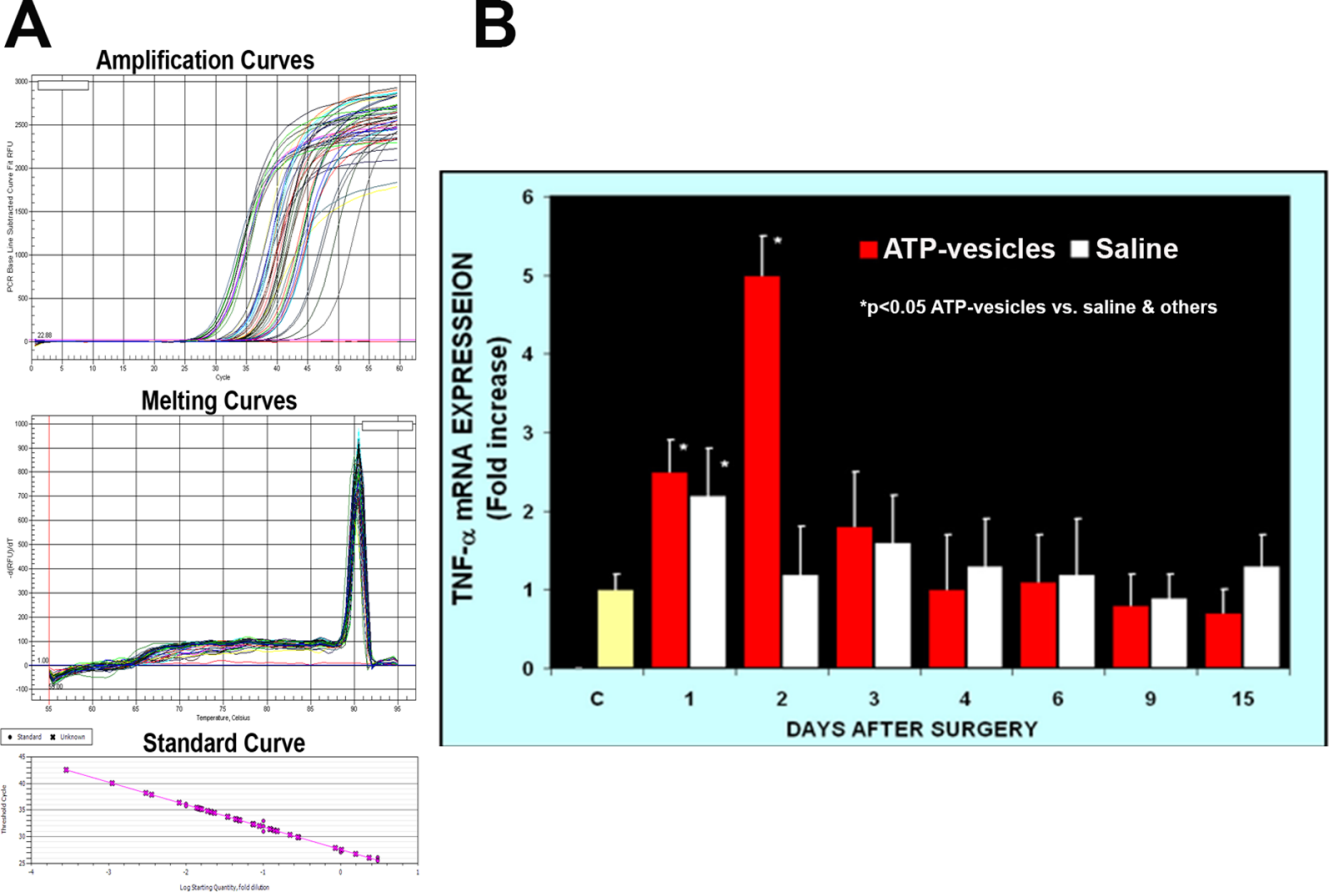
Real-time PCR comparison of TNF-α expression in wound tissues of ATP-vesicle–treated (study) and the saline-treated (control) groups. **(A)** represents the amplification curves, melting curves, and standard curve of real-time PCR and **(B)** shows their quantitative fold changes indicating 4-time higher expression at day 2 by ATP-vesicles. Data shown are the mean ± SD of four to six different wound tissues, with triplicate assays for each sample. Normal rabbit ear skin was used as baseline control (C). *Significantly different when compared with the saline-treated control group and the baseline control, p < 0.05.

Activated platelets and platelet microparticles are known to secrete numerous inflammatory mediators, which may or may not be related to hemostasis (Scull et al., 2010). MCP-1, IL-1β, and TNF-α are a few that have significant roles in wound healing. We used real-time fluorescence-based quantitative PCR to measure the expressions of MCP-1, IL-1β, and TNF-α mRNAs in these wound tissues from day one through day 15 after surgery and treatment. The expression levels in normal rabbit skin (without surgery or any treatments) were used as the baseline levels. The response in the normal saline-treated wounds indicated that MCP-1 expression increased at day 1 after surgery; however, wounds treated with ATP-vesicles showed an 9-fold increase in the MCP-1 expression level compared to the baseline level and a 2-fold increase compared to expression in the normal-saline–treated group (**Figures 1C**, **E**). MCP-1 immunostaining indicated higher expression in the ATP-vesicle–treated wounds than in the controls (**Figure 1D**), in agreement with the mRNA expression results (**Figure 1E**). The normal saline-treated group showed slight increases in IL-1β expression on day 1 after surgery, and these levels declined to baseline from day 2 until day 15 after surgery. By contrast, the ATP-vesicle–treated group showed significantly increased IL-1β expression at day 1 and peaked at day 3 after surgery at levels almost 4 times higher than that observed in the normal-saline–treated group (**Figure 2**). Similarly, the expression of TNF-α mRNA in the control group increased at day 1 after surgery, and then decreased to the baseline after day 1. The ATP-vesicle–treated group showed increased TNF-α expression at day 1 and a peak at day 2 after surgery and this expression was four times higher than the control group (**Figure 3**).

### Histology and Immunohistochemistry Studies

**Figure 4:**
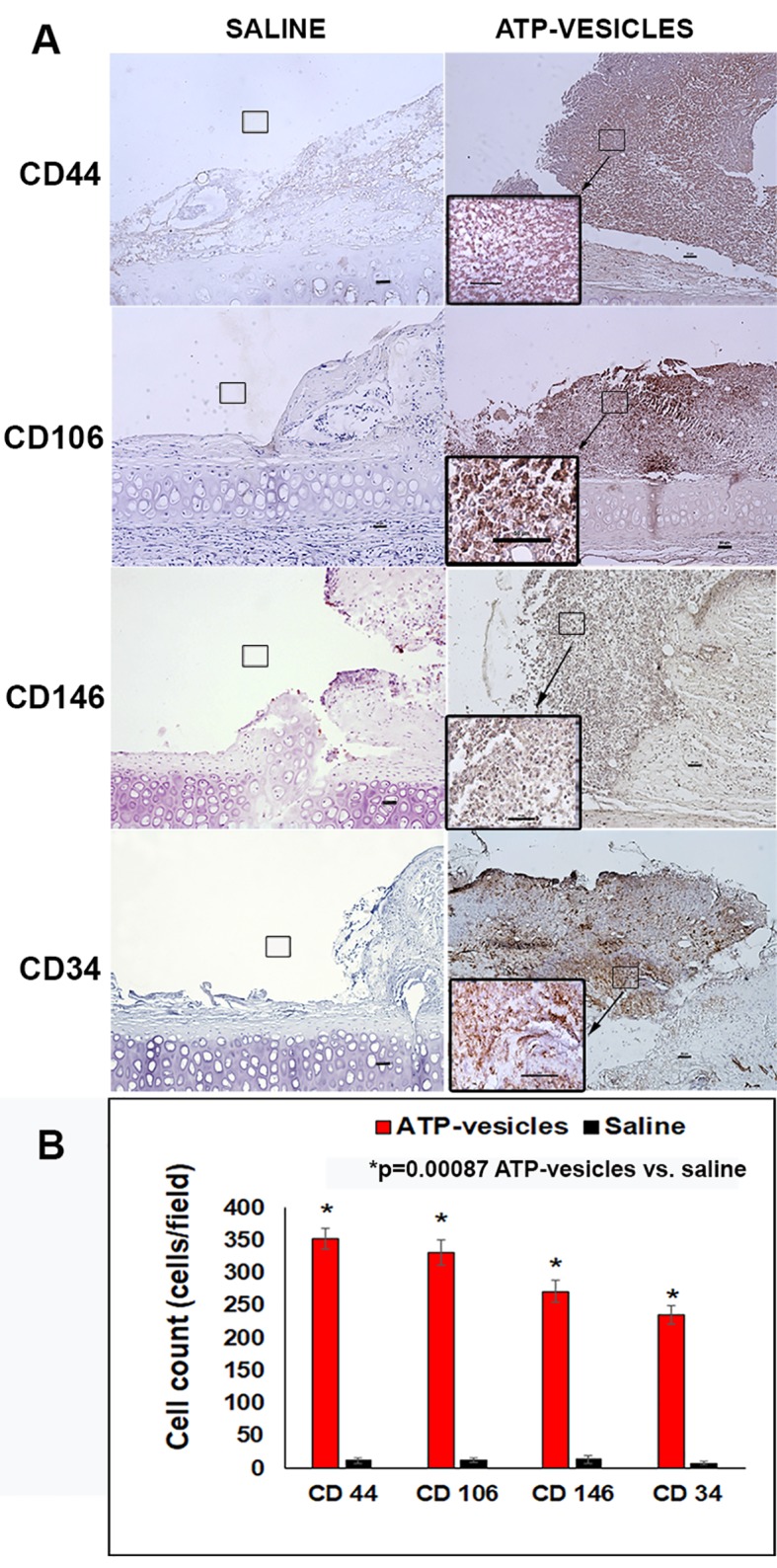
**(A)** Immunohistochemical staining of stem cell markers in wounds 24 hours after surgery. A comparison of tissue samples showing substantial granulation tissue growth in the ATP-vesicle–treated wounds, whereas the wounds treated with normal saline show almost no growth. The ATP-vesicle–treated wounds show significant numbers of positive CD44, CD104, CD146, and CD34 stained stem cells among the basal, interior, and superficial areas of the granulation tissue. Despite the numerous CD34-positive cells in the ATP-vesicle–treated wounds, a mature vascular system has not yet formed. The brown color indicates DAB-positive immunostaining (scale bar = 50 μm). **(B)** Graphical representation of the cells positive for CD44, CD104, CD146, and CD34 between the groups.

**Figure 5:**
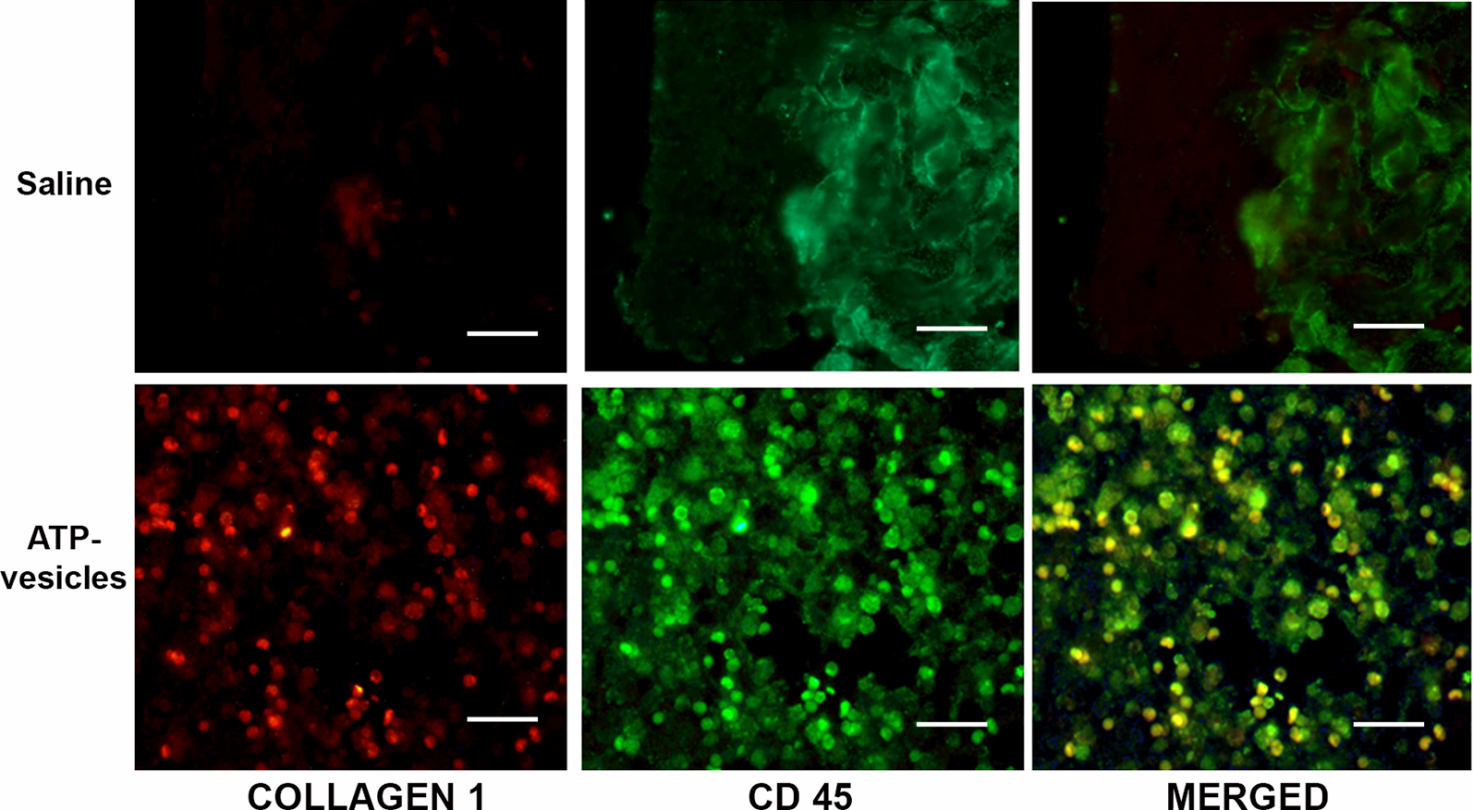
Co-localization study with CD45 and collagen type I antibodies at day 3. Positive collagen I (red) and CD45 (green) stainings were observed in the ATP-vesicle–treated wounds, demonstrating neovascularization and matrix formation in these wounds as early as day 3 after surgery (scale bar = 50 μm). Saline-treated wounds didn’t show any growth.

Neovascularization appeared to start within 24 h of surgery in the ATP-vesicle–treated wounds, which showed a significant increase in MSCs among the basal, interior, and superficial cells of the granulation tissue, as indicated by immunostaining for several markers, including CD44, CD106 (VCAM), perivascular marker CD146, and hematopoietic markers CD34 and CD45 (**Figures 4** and **5**). A large number of cells positive for CD44, CD106 (VCAM), CD146, CD34, and CD45 were detected in the ATP-vesicle–treated wounds, even though the mature vascular system had not yet formed. The saline–treated wounds showed scarcely any granulation tissue growth at the wound site at early days, so no positively stained cells were observed. The dual staining of collagen type I and CD45 in the ATP-vesicle–treated wounds indicated early extracellular matrix formation and neovascularization, which are required for wound closure, but no similar staining was observed in the control wounds (**Figure 5**).

**Figure 6:**
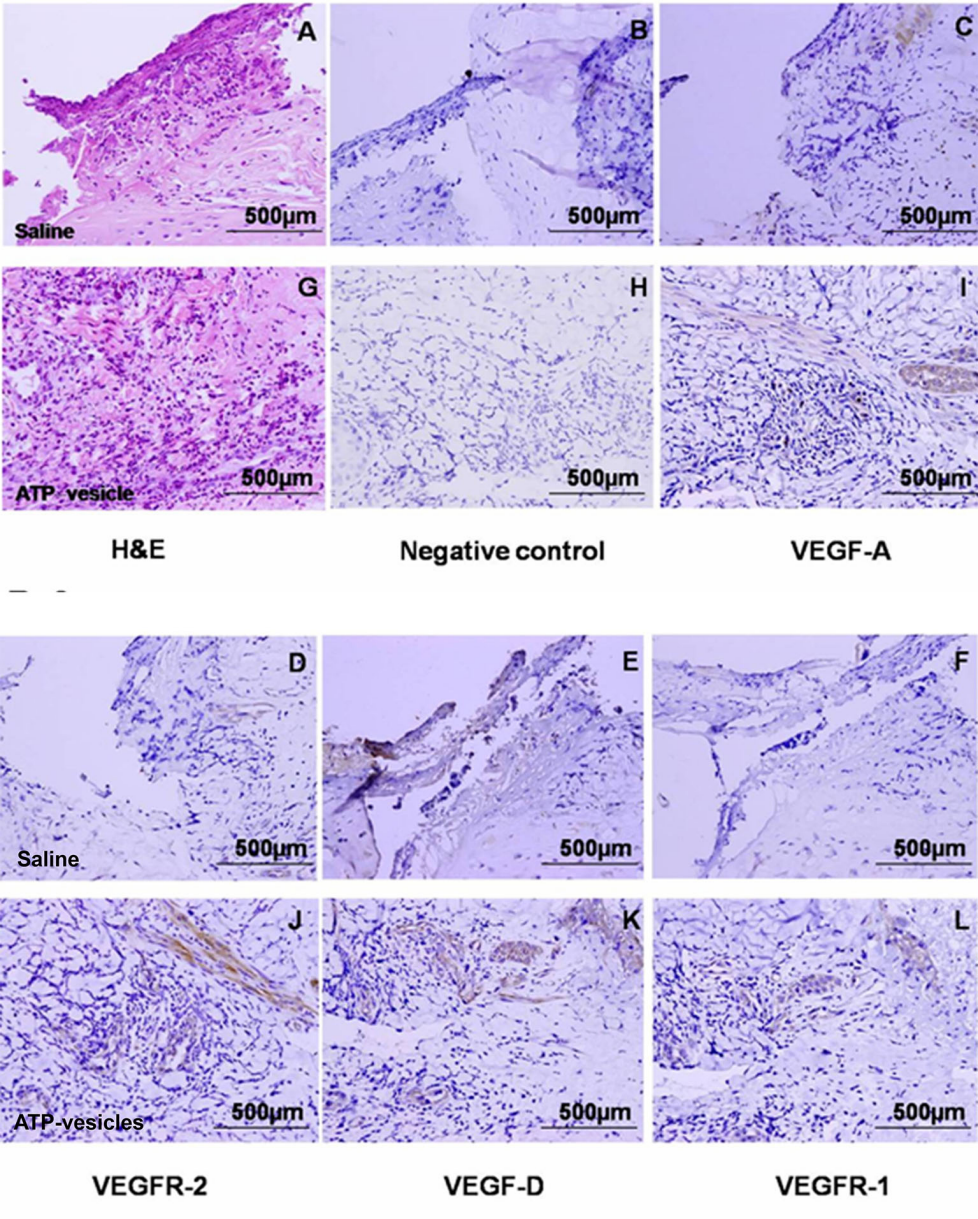
Immunohistochemical staining of VEGFs and their receptors in wound tissues at day 6 after surgery. The expression of VEGF-A, VEGFR-2, and VEGF-D were significantly higher in the ATP-vesicle–treated groups **(I**, **J**, and **K)** than in the normal-saline–treated groups **(C**, **D**, and **E)** after 6 days of treatment. VEGFR-1 also showed some increase in ATP-vesicle-treated wounds **(L)** as compared with that in saline-treated wound **(F)**. **(A** and **G)** are hematoxylin & eosin stainings; **(B** and **H)** are negative controls. The brown color indicates DAB-positive immunostaining (scale bar = 500 μm).

**Figure 7:**
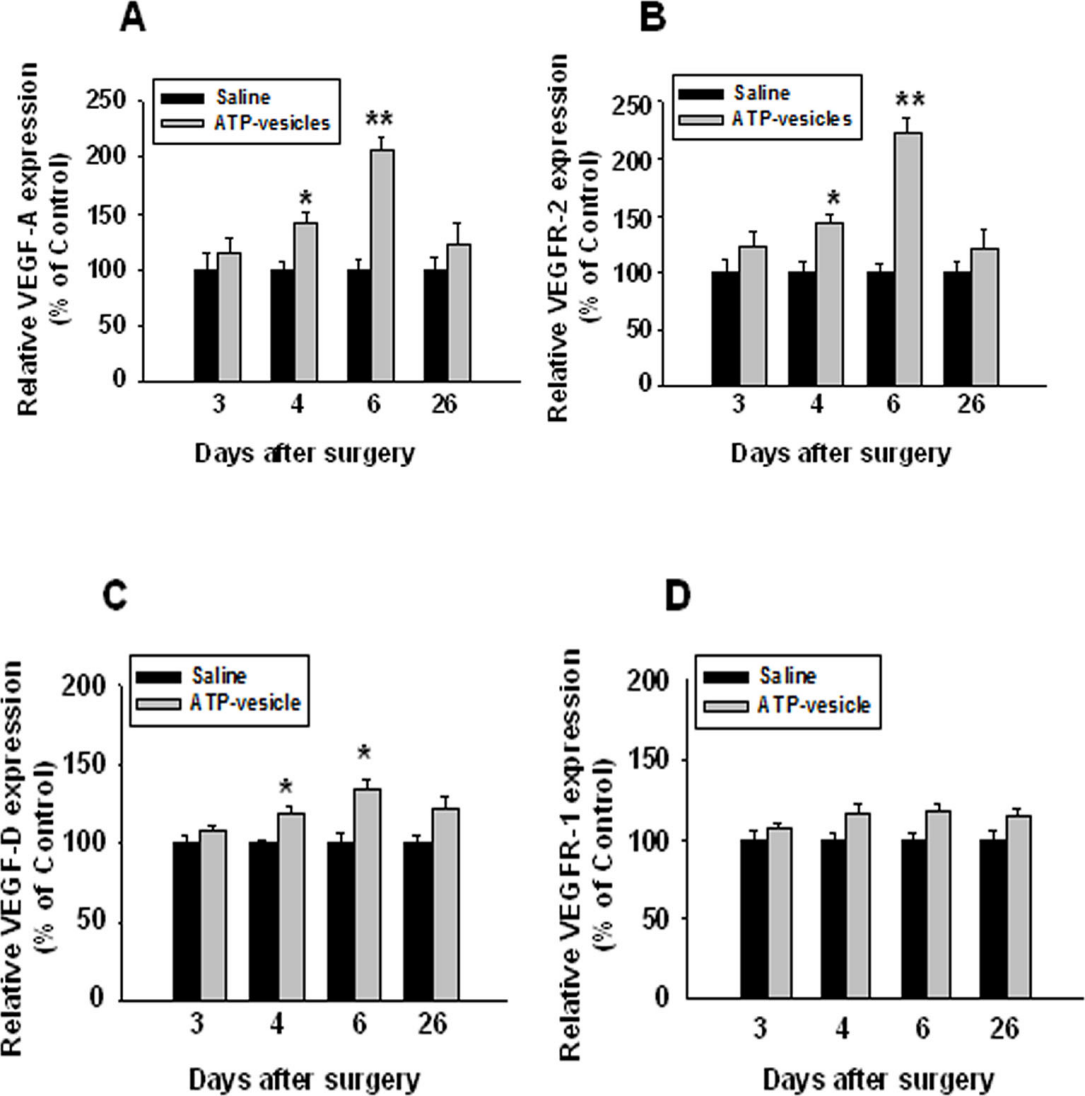
Immunohistochemical staining of VEGFs and their receptors in ATP-vesicle-treated and saline-treated wounds. The number of positively stained cells and total cells per high power microscopic field were counted. The increased expressions of VEGF-A, VEGF-D, and VEGFR-2 were clearly seen in ATP-vesicle-treated than saline-treated wounds **(A**, **B** and **C)**, but the increase of VEGFR-1 was not statistically significant **(D)**. The expression level of VEGFs and their receptors in each sample was expressed as a percentage of the total number of cells that are positively stained. The relative expression levels in ATP-vesicle-treated wounds was normalized to that of normal saline-treated wounds at the same time point. Data shown are the mean ± SD of four to six different wound samples, with at least 5 fields for each sample. Significant difference as compared to that of normal saline-treated group, *p < 0.05; **p < 0.01.

We further studied the mediators related to vascular regeneration by examining the growth factor profile changes in response to intracellular ATP delivery. The results were remarkable: In the H&E and immunohistochemical stained wound tissues at day 6 post surgery (**Figure 6**) the saline–treated wounds showed no changes in the expression of VEGF-A, VEGF-D, VEGFR-1, and VEGFR-2 throughout the treatments time, whereas the ATP-vesicle-treated wounds showed a significant upregulation of VEGF-A, VEGFR-2, and VEGF-D at days 4 and 6 after surgery (**Figures 7A–C**). By contrast, VEGFR-1 expression did not show any statistically significant change with any treatments (**Figure 7D**).

## Discussion

The problem of treating chronic wounds is rising swiftly due to the aging of the human population, a sharp increase in diabetes and obesity, and increasing health care costs. Wound healing is the consequence of an enormous interrelated biological events that are coordinated in response to injury and the wound microenvironment. More than 100 factors could be involved in the failure of wounds to heal (Brem and Tomic-Canie, 2007), but one critical pathophysiology is a deficient blood supply to the wound (Im and Hoopes, 1970). For many chronic wounds, ischemia may not be the initiating factor because most ulcers start from a combination of repeated trauma, neuropathy, pressure loading, infection, and/or trauma. However, tissue ischemia is the main cause that hinders healing. (Gottrup, 2004) Increasing the wound oxygen supply with hyperbaric oxygen therapy has not shown consistent improvement in healing (Berendt, 2006) because a lack of oxygen is only one part of ischemic pathophysiology—the most critical consequence of ischemia is a decrease in cellular energy supply (Im and Hoopes, 1970). Energy from ATP is essential in all phases of the wound healing, roughly consisting of protein building by amino acids, cell migration and proliferation, membrane transport, and growth factor production. Clinical trials of growth factors such as bFGF have shown effectiveness in non-ischemic wounds, but their effects disappear in hypoxic dermal ulcer models (Harding et al., 2002).

Finding the exact mechanisms underlying the rapid wound healing with the intracellular ATP delivery approach is challenging in the following five aspects because they are totally different from traditional wound healing process: 1) traditional wound healing process has a 3–6 days of lag time. However, our extremely rapid granulation tissue regeneration has totally eliminated this lag time; 2) in traditional healing process, monocytes/macrophages typically appear in the wound at 48–72 h after injury, but in our study, macrophage accumulation peaked at 24 h after surgery and these cells showed *in situ* proliferation, as well as an early polarization into the M2 subtype (Chiang et al., 2007; Wang et al., 2010; Howard et al., 2014; Howard et al., 2014; Sarojini et al., 2017); 3) in the traditional wound healing process, the wound cavity is first filled with a provisional extracellular matrix (fibrin clot), which is gradually replaced by granulation tissues; however, in our study, the early massive cell accumulation in response to ATP-vesicles seemed to morph directly into extracellular matrix; 4) in traditional wound healing process, reepithelialization goes through the top of the granulation tissue. In our study, because of the rapid granulation tissue growth, reepithelialization tunnels through the granulation tissue (Howard et al., 2014); and 5) traditional wound healing relies on fibroblasts to produce collagen in later phase, but our results have shown that accompanying the massive accumulation of macrophages, there is also a macrophage direct collagen production to transform the cell mass directly into extracellular matrix. All these findings are only seen in the wounds treated by ATP-vesicles and are not reported in the past with any other treatment. The results obtained from this study are very unique. Although they cannot explain all the unprecedented results, they at least provide some clues to the unique findings after using intracellular ATP delivery. The following factors are clearly shown their effects on these new phenomena.

### The Role of Platelets

**Figure 8:**
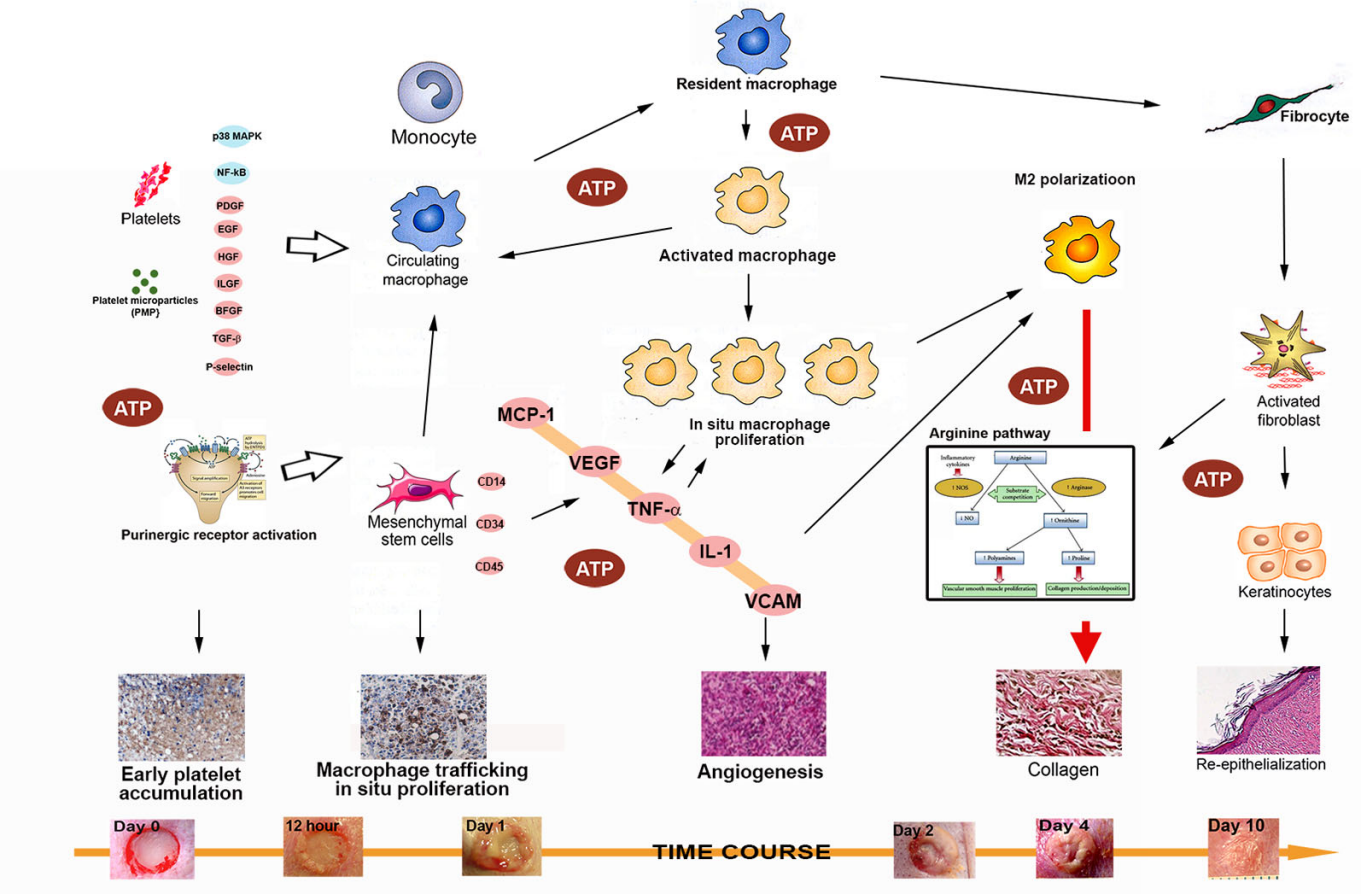
A schematic diagram of the possible mechanisms involved in the rapid tissue regeneration induced by intracellular ATP delivery. The following factors/pathways appear to play a major role in this process: 1). Early and rapid platelet activation and trafficking, with numerous cytokines and chemokines released to induce downstream actions; 2) Rapid macrophage trafficking, *in situ* proliferation, and early M2 polarization; 3) Massive release of cytokines and chemokines from activated macrophages, resulting in a reciprocal effect that further enhances the effects; 4) increases in levels of major cytokines and chemokines (MCP-1, TNF-α, IL-1β, and VCAM) enhance M2 polarization to facilitate direct collagen production and reepithelialization; 5) Enhanced mesenchymal stem cell accumulation and differentiation into multiple type of skin cells (keratinocytes, endothelial cells, pericytes, and monocytes) further accelerates the healing process; and 6) extracellular ATP activates purinergic receptors to provide additional enhancement of these processes.

Platelet adhesion to the extracellular matrix is the primary step in hemostasis, and the exposed wound collagen binds directly to platelet GP Ia/IIa and GP VI receptors (Yun et al., 2016). The ADP released from damaged endothelial cells and activated platelets are known to act on platelet P2Y1 and P2Y2 G protein coupled receptors (GPCR) and give rise to further platelet activation and release of ADP—a reciprocal reaction that probably can explain the very rapid platelet accumulation observed in the early hours following ATP-vesicle treatment. The downstream reactions of platelet activation cause monocyte activation and monocyte transformation into macrophages by at least two pathways: 1) platelet and platelet microparticles; and 2) the MCP-1 pathway. These reactions cause fast leukocyte (mainly monocyte/macrophage) migration and accumulation (**Figure 8**). At the same time, activated macrophages secrete MCP-1, which induces further cell accumulation. This type of reciprocal feedback cycle is the main cause for the exceptionally fast macrophage accumulation observed with the ATP-vesicle treatment. The intracellularly delivered ATP serves as the main energy source that keeps these cells alive and functioning at the very early stage where blood supply is still very limited.

### The Role of Cytokines, Chemokines, and Growth Factors

In this study, we found that wounds treated with normal saline showed some changes in several cytokines, but almost no changes in growth factors. The latter finding was difficult to explain because, in theory, growth factors should change during the healing process. A few previous reports have shown different results—some showed upregulation of various growth factors, whereas others did not show significant changes. However, almost all of these previous studies used growth factors or other stimulating agents, and these can have different stimulating effects on wound tissues (Corral et al., 1999). Our study provides energy to the cells thus the mechanisms are different. In the present study, although no growth factors were used, the expression of MCP-1 (CCL2), IL-1β, TNF- α, stem cell markers, vascular endothelial growth factors and receptors was markedly increased following ATP-vesicle treatment. This rapid and strong upregulation corresponded to the extremely fast migration and accumulation of inflammatory cells that, in turn, formed the early granulation tissue in the wound cavity.

Macrophage infiltration is regulated through chemokines, designated as C, CC, CXC, or CX3C chemokines depending on the number and location of the cysteine residues at their N terminus (Choi et al., 2016). CCL2 is also known as monocyte chemoattractant protein -1. The biological effect of MCP-1 is mediated through its interaction with its receptor, CCR2, which is expressed on mononuclear cells, vascular smooth muscle cells (VSMCs), and activated natural killer cells. Deficiency of either CCL2 or CCR2 impairs muscle regeneration. Furthermore, knockout (KO) of CXCL16 prevents macrophage accumulation in injured muscle and blocks muscle regeneration (Zhang et al., 2009).

One unique phenomenon observed in the present study is the very early and significant MCP-1 expression in the wounds treated with ATP-vesicles. MCP-1, as an IFN-γ-inducible chemokine (Bosco et al., 2004), is endowed with chemotactic and activating properties for macrophages and other leukocytes and is critically involved in the regulation of inflammatory processes. Interestingly, hypoxia induces a selective inhibition of macrophage MCP-1 production, which results in decreased gene transcription and reduced mRNA stability. Cell reoxygenation reverses these hypoxia-induced inhibitory effects within 24 h and restores MCP-1 mRNA expression (Bosco et al., 2004). Delivery of extracellular ATP can also stimulate MCP-1 production. It has been shown that both P2X and P2Y mediate the rapid release of proinflammatory cytokines and MCP-1 when extracellular nucleotides are released (Placido et al., 2006).

Previous studies have shown that inhibition of MCP-1 represents a negative regulatory mechanism for controlling macrophage-mediated leukocyte recruitment and that this inhibition significantly delays wound healing, particularly in reepithelialization, angiogenesis, and collagen synthesis (Bosco et al., 2004). Enhancement of macrophage accumulation *via* MCP-1 is also observed to occur independent of circulating monocytes. The many functions of MCP-1 in the wound healing process are not yet completely elucidated or they remain controversial, but years of studies have shown that MCP-1 has the following functions, which may contribute to the phenomena we have observed in our study:

1. Macrophage trafficking, proliferation, and polarization. This has been reported with respect to MCP-1 numerous times in the past, in many species, including mice, rats, pigs, and humans. A few reports have also appeared on rabbits, but most are related to atherosclerosis. A lower MCP-1 content in aged animals appears to contribute to a slow wound healing process (Shallo et al., 2003). Exogenous MCP-1 can also increase monocyte/macrophage recruitment, collateral vessel formation, and blood flow to the tissue in ischemic models (Niu et al., 2013). MCP-1 influences the effector state of macrophages and other cell types, and local overexpression of MCP-1 can induce infiltration by macrophages.
2. MCP-1 is one of the chemokines most often observed after tissue ischemia, and it has been measured to be one of the principal angiogenic factors associated with the recruitment of monocytes. Monocyte cells increase neovascularization by discharging proangiogenic mediators and/or by trans-differentiation into endothelial-like cells. In wound healing, MCP-1 also has the function of stimulating collagen expression *via* endogenous upregulation of TGF-beta and MMP expression in stimulated fibroblasts (Becker, 2005), thereby enhancing collagen fiber formation.
3. MCP-1 promotes endothelial cell proliferation and angiogenesis in animals and humans by several mechanisms. It is known that TGF-beta promotes the formation of new blood vessels, and this is mediated by MCP-1, which modulates the angiogenic effect of TGF-beta by recruiting VSMCs and stem cells toward endothelial cells.

Macrophages are known to produce numerous cytokines and chemokines. Although resting macrophages only produce low levels of proinflammatory cytokines, they produce a large number of cytokines, including MCP-1, IL-1, IL-6, IL-12, TNF-beta, and iNOS, after stimulation by processes such as wounding (Koh and DiPietro, 2011). All these mediators contribute to the enhanced healing process but the production of MCP-1 by activated platelets and the ability of macrophage to produce MCP-1 results in another reciprocal effect that can partly explain the extremely rapid macrophage trafficking, *in situ* proliferation, and M2 polarization. The increased expression of other cytokines in our wound samples corresponds well with increased macrophage accumulation, and the subsequent release of cytokines further enhances the wound healing process in our rabbits.

IL-1β is a member of the interleukin 1 family of cytokines. However, when compared with MCP-1, the function of IL-1β in wound healing is less well studied. IL-1β is an important early inflammatory mediator secreted by a range of cells, including neutrophils, epithelial cells, and macrophages (including M1 and M2), and its major effect is pleiotropic expression of growth factors. Acute inflammation arises during the initial stage of wound healing. Secretion of IL-1β within the wound bed after an injury can stimulate the creation of secondary inflammatory mediators, such as IL-6, IL-8, and prostaglandin E2 (PGE_2_). These mediators coordinates subsequent activity in the wound bed. In this study, IL-1β mRNA showed an increase in the first 24 h, followed by a sudden decrease, in normal saline treated wounds, and this pattern fits some previous reports (Bai et al., 2008). However, the ATP-vesicle–treated group showed IL-1β increases from days 1 to 3 to levels four times higher than observed in the control group. After day 6, the level was even lower than the control wounds.

In rodent studies, IL-1β and COX-2 expression is upregulated relatively quickly and persists for at least 48 h after mucosal injury (Sandulache et al., 2007). Moreover, macrophages isolated from diabetic humans and mice wounds exhibit a proinflammatory phenotype, comprising expression and secretion of IL-1β, and this environment seems to be sufficient to inhibit the healing process. Inhibiting the IL-1β pathway using a neutralizing antibody in diabetic mice induced a switch from proinflammatory to healing-associated macrophage phenotypes, increased the levels of wound growth factors, and improved healing of these wounds (Mirza et al., 2015). The pattern of IL-1β rapid changes coincided well with rapid macrophage trafficking and accumulation during the inflammatory phase seems to contribute to the enhanced healing in the ATP-vesicle-treated wounds.

TNF-α is a cell cytokine involved in acute phase reaction of systemic inflammation. It is created mainly by the activated macrophages, even though it can be created by many other cell types, like CD4+ lymphocytes, NK cells, neutrophils, mast cells, eosinophils, and neurons. The expression of TNF-α in the present study differed somewhat from traditional findings. In normal wound healing, the highest the levels of TNF-α occur from 12 to 24 h after wounding (Mori et al., 2002). At that time, fibroblast migration starts and forms granulation tissues. A recent study by Ritsu et al. on mice (Ritsu et al., 2017) reported the detection of TNF-α synthesis just after wound creation, followed by an increase during the first several hours, a peak level was reached on day 1, and then decreased to the basal level. TNF-α, at low levels, will be able to promote wound healing by secondarily stimulating inflammation and increasing the levels of macrophage-produced growth factors. However, TNF-α at higher levels, especially for extended periods of time, has a detrimental effect on healing. TNF-α suppresses the synthesis of extracellular matrix proteins and TIMPs, while increasing the synthesis of Matrix metalloproteinase (MMP-1, MMP-2, MMP-3, MMP-9, MMP-13, and MT1-MMP). In addition, elevated levels of TNF-α also have a similar response to those of IL-1β.

In our study, the expression of TNF-α increased after surgery in the control group, in agreement with previous reports (Schafer and Werner, 2007). However, the increase observed in the ATP-vesicle-treated group at day 2 was more than two times higher than the control value. All the changes in cytokines occurred concomitantly with the rapid tissue regeneration, indicating their potential roles, although the exact contribution of each cytokine is not entirely clear. A reasonable assumption is that the increases in TNF-α, at a moderate level, have a positive effect on healing of rabbit dermal wounds.

Macrophages permeate into the wound bed mostly from the circulation and from neighboring unwounded tissues. Successful macrophage extravasation from the blood requires specific cell-cell adhesion molecules, including immunoglobulins such as VCAM, integrins, and P and E selectins. The upregulation of VCAM-1 in endothelial cells by cytokines results from increased gene transcription, such as that occurring in response to TNF-α and IL-1, and through stabilization of messenger RNA (mRNA) for cytokines such as IL-4. Functional tandem NF-κB sites are present in the promoter region of the VCAM-1 gene. In comparison with the previously mentioned cytokines, research on VCAM is relatively scarce, with only a few studies on atherosclerosis in the literature (Nakashima et al., 1998), and very little information is available on wound healing. Thus, our study is the only one that shows upregulation of VCAM (CD106) expression in rabbit wounds at 24 h after surgery, together with increased expression of CD44, CD146, and CD34. The 24-h time point is also the time when the highest macrophage trafficking occurs, and this fits with previous findings in aortic atherosclerosis in rabbits (Truskey et al., 1999). The result also agrees with the function of VCAM (CD106) in macrophage trafficking, as well as early angiogenesis in the wound healing process. A study using human dermal microvascular endothelial cell (HMEC) line by Xu et al. (1994) reported that TNF-α dramatically induced VCAM-l expression, which peaked at 4 h and was maintained through 24 h, remaining elevated until 72 h.

### The Role of MSCs and Growth Factors

Current knowledge indicates that MSCs can enhance wound healing through several mechanisms: 1) increase angiogenesis by secretion of proangiogenic factors, 2) increase M2 macrophage infiltration and polarization, 3) recruit endogenous stem/progenitor cells, 4) produce and remodel extracellular matrix, and 5) differentiate into endothelial cells, pericytes, fibroblasts, and keratinocytes (Motegi and Ishikawa, 2017).

MSCs can be deliberated as non-hemopoietin multipotent stem-like cells that are capable of differentiating into both mesenchymal and non-mesenchymal ancestries. However, no specific single marker clearly defines MSCs. In fact, at present, MSCs are identified through a combination of physical, phenotypic, and functional properties (Sasaki et al., 2008). Our immunohistochemistry stainings have identified several stem cell markers, such as CD44, CD106, CD146, CD34, and CD45, which are all upregulated in ATP-vesicle–treated wounds (as seen in **Figures 4** and **5**). The mechanism for their final contribution to extremely rapid tissue regeneration may be quite complicated, but even these known pathways are very significant, as are other mediators (**Figure 8**).

In wound healing, the formation of new blood vessels is essential to sustain the newly formed granulation tissue; thus, impaired wound repair is associated with a dramatic decrease in the formation of vascular endothelium (Kampfer et al., 2001). VEGF, bFGF, TGFβ, epidermal growth factor (EGF), keratinocyte growth factor (KGF), TNF-α, and interleukins are all reported to be involved in angiogenesis (Singer and Clark, 1999). However, only VEGF, bFGF, and PDGF have shown a direct mitogenic effect on vascular endothelial cells. In the present study, treatment with ATP-vesicles caused significant upregulation of VEGF and its receptors from day 4 to day 6 after surgery, which corresponded to the period of rapid granulation tissue formation and the initiation of neovascularization. Understandably, the normal saline-treated wounds did not show any increase in these growth factors.

Although our study indicated very early neovascularization, the tissues were not mature enough to provide a blood supply. During this “avascular” period, massive cell necrosis is quite common. However, necrosis was totally absent in our study. We hypothesize that our novel intracellular ATP delivery modality provides these cells with an adequate energy supply. This continuous intracellular energy supply not only maintains a smooth transition from early cell accumulation to later extracellular matrix formation, but it also prevents ongoing inflammatory reactions evoked by massive cell death.

ATP is a multifunctional nucleotide that plays an important role in cell biology not only as energy source, but also as a coenzyme and a signaling molecule (Knowles, 1980). During the wound healing process, ATP delivered intracellularly also stimulates the migration of inflammatory cells and bone marrow-derived progenitor cells from the circulation to accumulate in the wounds, and stimulates cells to synthesize and/or secrete more cytokines and growth factors to attract more cells, resulting in a beneficial reciprocal cycle.

We believe that the enhanced healing effect observed is mainly achieved by the supply of energy by the intracellular delivery of Mg-ATP, although certain supportive effects may also be contributed by other functions of ATP. Normal skin contains resident macrophages at a low density of approximately 1–2 per mm^2^ (Khmelewski et al., 2004). During wound repair, large numbers of monocytes are recruited into injured tissue, where they differentiate into macrophages, so that the density of macrophages increases to more than five times than in the normal tissue. The magnitude of this increase in these cells observed in our study is exceptional (Howard et al., 2014; Sarojini et al., 2017). The contribution of increases in VEGF family compounds is clearly seen in the later stage characterized by the transition from massive cell accumulation directly to extracellular matrix due to the rapid formation of microvasculature.

This study has some limitations. Wound healing is one of the most composite processes in human life, with over 100 known physiologic factors contributing to healing deficiencies in non-healing wounds as stated above (Brem and Tomic-Canie, 2007). We were able to detect early upregulation of IL-1β, TNF-α, MCP-1, and stem cells in ATP-vesicle–treated wounds. The gene expression data sets are in the form of upregulated mRNA transcription and increased protein translation of specific cytokines and stem cell protein markers. The data sets, supplement each other and confirm and consolidate the overall inference drawn that there is significant upregulation. Our group has recently published advances in RNA analysis by RNA array technique as well as RNA seq technology to obtain an unbiased evaluation of gene expression in rabbit cells in culture treated with ATP nanoliposomes or gamma-thio ATP nanoliposome control (Kotwal et al., 2018). However, many more cells and mediators are known to be involved in the healing process, and upregulation of these cytokines is only part of this rapid tissue growth. Many additional mechanisms need to be explored, but the data reported here represent the first step in delineating this complex and exciting process.

In conclusion, intracellular ATP delivery directly in wounds enhanced rapid growth. This phenomenon was accompanied by a very early platelet accumulation and MCP-1 upregulation and was followed by gradual upregulation of cytokine IL-1β, TNF-α, and stem cells, along with increases in vascular endothelial growth factor expression. This well-coordinated process results in enhanced wound healing, but without hypertrophic scar formation or any other unusual growth. Although many mechanisms are still unclear and some of them may be species specific, this new approach will be highly significant if it can be duplicated in human wound healing.

## Data Availability Statement

All datasets generated for this study are included in the article.

## Ethics Statement

The animal study was reviewed and approved by the University of Louisville Institutional Animal Care and Use Committee.

## Author Contributions

YM, HS, RW, QZ, and SC designed experiments, performed research and analyzed data. YM, HS, and SC wrote the manuscript. JW and SC performed animal experiments. SE contributed to immunohistochemistry results. YM, HS, GK, and SC provided critical edits to the manuscript.

## Funding

This work was supported in part by NIH grants DK74566, AR52984, OD021317, and DK105692 to SC.

## Conflict of Interest

SC is the founder of Noveratech LLC and holds patents related to ATP-vesicles and their use in wound healing. GK is the employee of Noveratech LLC.

The remaining authors declare that the research was conducted in the absence of any commercial or financial relationships that could be construed as a potential conflict of interest.
